# Blood-Brain Barrier: More Contributor to Disruption of Central Nervous System Homeostasis Than Victim in Neurological Disorders

**DOI:** 10.3389/fnins.2020.00764

**Published:** 2020-08-06

**Authors:** Minjia Xiao, Zhi Jie Xiao, Binbin Yang, Ziwei Lan, Fang Fang

**Affiliations:** ^1^Department of Neurology, Second Xiangya Hospital, Central South University, Changsha, China; ^2^Department of Critical Care Medicine, The First Affiliated Hospital, College of Medicine, Zhejiang University, Hangzhou, China

**Keywords:** blood-brain barrier, acute ischemic stroke, intracerebral hemorrhage, Alzheimer’s disease, Parkinson’s disease, multiple sclerosis

## Abstract

The blood-brain barrier (BBB) is a dynamic but solid shield in the cerebral microvascular system. It plays a pivotal role in maintaining central nervous system (CNS) homeostasis by regulating the exchange of materials between the circulation and the brain and protects the neural tissue from neurotoxic components as well as pathogens. Here, we discuss the development of the BBB in physiological conditions and then focus on the role of the BBB in cerebrovascular disease, including acute ischemic stroke and intracerebral hemorrhage, and neurodegenerative disorders, such as Alzheimer’s disease (AD), Parkinson’s disease (PD), and multiple sclerosis (MS). Finally, we summarize recent advancements in the development of therapies targeting the BBB and outline future directions and outstanding questions in the field. We propose that BBB dysfunction not only results from, but is causal in the pathogenesis of neurological disorders; the BBB is more a contributor to the disruption of CNS homeostasis than a victim in neurological disorders.

## Introduction

The neurovascular unit (NVU) is a functional complex critical to the stability of the CNS microenvironment and is composed of endothelial cells (ECs), glial cells, pericytes (PCs), neurons, and the extracellular matrix (ECM). Communication between the individual NVU components is key for its correct function. The blood-brain barrier (BBB) is considered the core structure of the NVU (Saint-Pol et al., 2020). It is established by tightly sealed ECs located at the luminal surface of the brain’s vascular tree (Sweeney et al., 2019b). The presence of cell surface proteins, ion channels, efflux pumps, enzymes, specific receptors, and transporters on pericytes (PCs), vascular smooth muscle cells (VSMCs), and ECs maintain BBB integrity (Sweeney et al., 2019b) and allow bidirectional regulation of substances by transcellular or paracellular transport (Haseloff et al., 2015; Xiaoyan et al., 2018).

Under normal conditions, ECs in the CNS microvasculature have fewer endocytic vesicles than those in the endothelia of the peripheral capillaries and only ensure the supply of basic nutrients, including oxygen and glucose, to the neurons (Persidsky et al., 2006). Thus, decreased transcytosis across the BBB restricts permeability and impedes the delivery of therapeutic agents, which is of great importance for restoring CNS homeostasis under pathological conditions (Liebner et al., 2018). Additionally, ECs also bidirectionally communicate with surrounding cells to maintain the dynamic nature of the BBB.

Blood-brain barrier dysfunction is a pathophysiological hallmark in the pathogenesis of multiple CNS diseases (Cummins, 2011). Extensive evidence implicates it in cerebrovascular, neurodegenerative, and neuroinflammatory diseases. Here, we first illustrate the physiological structure of the BBB and then discuss the development of junctional complex perturbations in pathological conditions, including cerebrovascular disease and neurodegenerative disorders. Finally, we summarize recent advancements in therapeutics targeting brain-barrier function. We emphasize the significance of the BBB in neurological disorders and put forward crucial questions to be answered in the future.

## The Blood-Brain Barrier Establishment

### Angiogenesis and Barriergenesis

Prior to BBB development, neuroectodermal neural progenitor cells secrete vascular endothelial growth factor (VEGF) to guide angioblasts from the perineural vascular plexus across the neuroectoderm to initiate angiogenesis (Potente et al., 2011; Obermeier et al., 2013). Meanwhile, neural progenitor cells also release Wnt ligands and activate the Wnt/β-catenin signaling pathway, which induces expression of genes indispensable for angiogenesis and BBB barriergenesis (Stenman et al., 2008). Orphan G-protein-coupled receptor 124 (Gpr124), a coactivator of the Wnt/β-catenin pathway, also impacts angiogenesis (Zhao et al., 2015a). During angiogenesis, nascent vascular ECs secrete platelet-derived growth factor-BB (PDGF-BB) and recruit PCs via the PDGF-receptor (PDGFR) (Sweeney et al., 2016, 2019b). PCs work synergistically with vascular ECs, deliver basement membrane (BM) proteins, and regulate integration of the BBB through interaction with the astrocyte end-feet that encircle vessels (Daneman et al., 2009; Armulik et al., 2010). The Sonic hedgehog (Hh) signaling pathway is crucial for EC polarity. Sonic hedgehog (SHH) released by astrocytes combines with Hh expressed by ECs to promote the expressions of tight junction (TJ) proteins and junctional adhesion molecules (JAMs). The SHH signaling pathway also suppresses the expression of proinflammatory factors and intercellular adhesion molecule-1 (ICAM-1), thereby inhibiting infiltration of leukocytes (Alvarez et al., 2011; Langen et al., 2019; Sweeney et al., 2019b).

### Anatomical Structure

Endothelial cells in the CNS are distinct from those other tissues due to the following properties: possession of continuous intercellular TJs, lack of fenestration and pinocytosis, and low expression of leukocyte adhesion molecules (Obermeier et al., 2013; Langen et al., 2019). The BBB formed by the ECs is a dynamic but impassable wall, restricting the exchange of paracellular or transcellular substances and abrogating the infiltration of leukocytes.

Tight junction proteins, JAMs, adherens junctions (AJs), and gap junctions are responsible for restricting substance movement by prohibiting the paracellular pathway (Saint-Pol et al., 2020). On the other hand, enzymes and influx/efflux pumps maintain the dynamic nature of the barrier, limiting non-specific transport while also allowing specific transport (Saint-Pol et al., 2020).

Junctional molecules at the BBB are divided into four categories: TJ (with TJ proteins and TJ-associated proteins), AJ, JAM, and gap junctions (Chow and Gu, 2015; Tietz and Engelhardt, 2015; Sweeney et al., 2019b). They communicate with the cellular actin cytoskeleton via membrane-associated guanylate kinases (MAGUK), such as zonula occludens-1 (ZO-1), ZO-2, and ZO-3 (Bazzoni and Dejana, 2004; Zlokovic, 2008). TJ proteins are closest to the apical membrane and seal the BBB, thus acting as potential therapeutic targets. On the other hand, AJs, composed mainly of VE-cadherin and platelet endothelial cell adhesion molecule-1 (PECAM-1), are closest to the basolateral membrane (Sweeney et al., 2019b). JAMs consist of proteins, including JAM-A, JAM-B, and JAM-C, and endothelial cell adhesion molecules (ESAMs), and regulate transmigration of leukocytes across the BBB (Garrido-Urbani et al., 2014; Sweeney et al., 2019b). Gap junction proteins include connexin-37, connexin-40, and connexin-43 and act as a sort of “hemichannel” to facilitate communication between adjacent ECs (Sweeney et al., 2019b).

Claudin-1 is a non-specific claudin in vessels, overexpression of which has been reported to reduce the disease burden in the chronic phase of a mouse model of multiple sclerosis (MS) (Pfeiffer et al., 2011). However, it has also been shown that claudin-1 overexpression decreased the level of claudin-5 and induced a proinflammatory phenotype in ECs in chronic stroke (Sladojevic et al., 2019). This suggests that claudin-1 overexpression compensated for BBB dysfunction only in the acute phase of injury. In addition to this, genetic polymorphisms in claudin-1 have been shown to contribute to small vessel vascular dementia (Srinivasan et al., 2017). Claudin-3 is involved in BBB induction and maintenance through signaling via the Wnt/β-catenin pathway (Polakis, 2008), while claudin-5 restricts the entrance of molecules up to 800 Da (Morita et al., 1999). Genetically modified mice lacking claudin-5 demonstrated leakage of intravascular tracers less than 800 Da in size despite having an intact BBB; 95% of pharmaceuticals are within this size range, implying that modulation of claudin-5 could be a potential target for drug delivery (Tsukita et al., 2003; Haseloff et al., 2015). Castro Dias et al. (2019) found that mice lacking claudin-12 still maintained an intact BBB as claudin-12 appears to be more important for cardiovascular functions (Castro Dias et al., 2019). It has also been shown to be involved in paracellular Ca^2+^ permeation in enterocytes (Saitou et al., 1997). To date, the exact function of claudin-12 in the CNS remains unknown and needs further investigation. Conversely, occludin is specifically expressed by the cells of the BBB rather than in non-neuronal tissues (Daneman et al., 2010a). Consequently, occludin knock-out mice show morphologically normal TJs despite development of brain calcification (Fujita et al., 2008). LSR/angulin-1 are significant for maintenance of BBB characteristics, so loss of LSR results in leakage of small molecules (Ikenouchi et al., 2005; Sohet et al., 2015).

Cadherins and PECAM-1 are examples of proteins that make up AJs. VE-cadherin is important for AJ assembly and BBB regulation (Li et al., 2018). In conditions of oxygen-glucose deprivation (OGD), VE-cadherin internalization induces BBB hyperpermeability via the RhoA/ROCK2 pathway (Chen J. et al., 2019). VE-cadherin phosphorylation under pathological conditions results in uncoupling of ECs and increased vascular permeability; thus, it may also serve as a novel target in moderating BBB dysfunction (Li et al., 2018).

ZO-1 is indispensable for assembly of junction complexes between ECs. Both downregulation and phosphorylation of ZO-1 results in impaired BBB integrity (Chen W. et al., 2019). Connexin is a hemichannel protein expressed by ECs. It opens at low extracellular concentrations of Ca^2+^ or under OGD and perturbs transport across the BBB (Tachikawa et al., 2020). Knockout of Connexin-43/30 in astrocytes (ACs) results in end-feet edema and loss of aquaporin-4 (AQP-4) expression (Ezan et al., 2012).

JAM-A is a unique protein in that it has diverging functions under physiological and pathological conditions. During homeostasis, it moderates paracellular transport, and during inflammatory conditions, it is redistributed to the apical surface of ECs and serves as an adhesion molecule, regulating leukocyte migration (Stamatovic et al., 2012). It has been shown that JAM-A antagonists can modulate leukocyte infiltration in ischemia/reperfusion (I/R) injury, thereby making JAM-A a potential therapeutic target (Sladojevic et al., 2014). Mice void of JAM-B show extensive vacuolation in the brain parenchyma along with neurodegeneration (Schottlaender et al., 2020). Moreover, bi-allelic JAM2 variants result in brain calcification, which could be attributed to dysfunction of solute transport (Schottlaender et al., 2020).

ECs attach to the BM via the binding of integrins to ECM ligands and stimulate multiple pathways, including growth factors, growth factor receptors, or transactivation of growth factor receptors. They have Na^+^-K^+^-Cl^–^ cotransporters and Na^+^/H^+^ exchangers at the luminal side and Na^+^/K^+^-ATPases at the abluminal side (Baeten and Akassoglou, 2011).

Anatomically, PCs share a common BM and interdigitate with ECs, forming peg-and-socket contacts in regions lacking a BM via *N*-cadherin and connexins (Winkler et al., 2011); they have multiple functions, including adjusting vascular stability (Armulik et al., 2011), managing capillary diameter to regulate cerebral blood flow (CBF) (Hall et al., 2014), and phagocytosing toxins (Sagare et al., 2013) to ensure BBB stability (Winkler et al., 2011). PC coverage of ECs is mediated through release of PDGF-β and activation of PDGFR by ECs. *Pdgfrb^–/–^* mice manifest with a low pericyte coverage rate, aberrant capillary dilation, and increased BBB permeability (Daneman et al., 2010b).

Astrocytes are the most abundant glial cells in the CNS (Liu and Chopp, 2016). Their functions include balancing the extracellular potassium concentration, regulation of neurotransmitter delivery, excretion of growth factors, and metabolic support to neurons (Gee and Keller, 2005). Secretion of SHH, retinoic acid (RA) and angiopoietin-1 (Ang-1) by ACs is crucial to sustain EC impermeability (Alvarez et al., 2011; Gurnik et al., 2016). In turn, ECs can regulate differentiation of ACs via production of bone morphogenetic proteins (BMPs) and activating the Smad signaling pathway in progenitors (Imura et al., 2008).

ECs, ACs, and PCs all secrete ECM and form the BM. Multiple structural proteins (for instance, collagen type 4 and laminin), cell adhesion molecules, and extracellular matrix proteins make up the BM (Carvey et al., 2009). The BM interacts with surrounding cells to control vascularization and coordinate signaling between components of the NVU (Obermeier et al., 2013).

### Transport System

**FIGURE 1:**
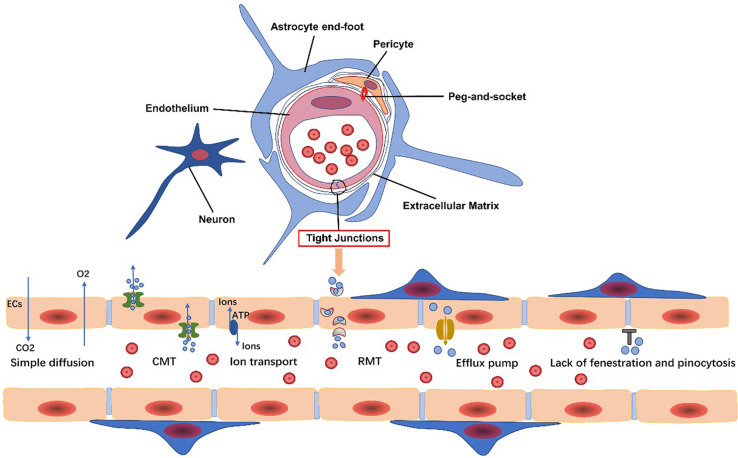
Endothelial cells, pericytes, the extracellular matrix, glial cells, and neurons are all basic components of the neurovascular unit. The blood-brain barrier (BBB) is composed of specialized endothelial cells. The transport system of the BBB includes carrier-mediated transport (CMT), receptor-mediated transport (RMT), efflux transporters, and ion transport. Gases, such as oxygen and carbon dioxide, and small lipophilic molecules less than 400 Da in size can freely diffuse across the BBB.

The BBB transport system can be classified into the following categories: carrier-mediated transport (CMT), receptor-mediated transport (RMT), efflux transporters, and ion transporters (Sweeney et al., 2019b) (Figure 1). Corresponding transporters to these can also be found on PCs. Each transport system is discussed in turn, and then, we elaborate several common transporters involved in undermentioned diseases.

Carrier-mediated transport is responsible for transport of solutes, including carbohydrates, amino acids, and so on. Glucose transporter type 1 (GLUT1) is critically important in providing the CNS with energy. Therefore, GLUT1 dysfunction contributes to a variety of CNS diseases, termed GLUT1-deficiency syndromes (Glut1-DS), such as drug-resistant epileptic seizures, psychomotor retardation, ataxia, and microcephaly (Galochkina et al., 2019; Koch and Weber, 2019). It also plays a role in AD pathogenesis as discussed below.

The main efflux pumps are the ATP-binding cassette (ABC) transporters. ABC transporters utilize ATP and pump out xenobiotics or endogenous metabolites to maintain EC homeostasis (Chow and Gu, 2015; De Rosa et al., 2015; Miller, 2015). ABCB1 (also known as *P*-glycoprotein, *P*-gp), ABCG2, and ABCC1 are all efflux transporters involved in the elimination of amyloid-β (Aβ), and so inhibition of these influences the Aβ level in the brain (Shubbar and Penny, 2020). In addition to AD, *P*-gp is involved in pathogenesis of stroke, epilepsy, and MS through upregulation or downregulation of expression influencing the inflammatory response (Huang et al., 2019).

Low-density lipoprotein receptor–related protein (LRP) is a lipoprotein receptor. It efficiently mediates clearance of Aβ, particularly Aβ42, via an LRP1/apolipoprotein E (APOE) isoform-specific mechanism (Ma et al., 2018). Both *P*-gp and LRP are regulated by phosphatidylinositol-binding clathrin assembly protein (PICALM) and PICALM deficiency diminishes transcytosis and clearance of Aβ (Zhao et al., 2015a; Storck et al., 2018). Conversely, the receptor for advanced glycation end products (RAGE) interacts with Aβ and mediates its transport across the BBB, thus inducing the release of proinflammatory cytokines (Deane et al., 2003).

Na^+^-K^+^-ATPases, Na^+^-K^+^-2Cl^–^ cotransporters (NKCC1), Na^+^-H^+^ exchangers (sodium pump), and Na^+^-Ca^2+^ exchangers are vital transporters for ionic equilibrium. Na^+^-K^+^-ATPase and NKCC1 function to maintain balance of sodium-potassium concentrations in the brain to guarantee stable physiological activity of neurons. The Na^+^-H^+^ exchanger is key for regulating normal pH of ECs, and the Na^+^-Ca^2+^ exchanger maintains a low level of intracellular Ca^2+^ in ECs (Sweeney et al., 2019b). Dysfunction of ion transporters contributes to ionic imbalance, resulting in release of inflammatory mediators.

## Cerebrovascular Disease

### Acute Ischemic Stroke

Acute ischemic stroke (AIS) is primarily caused by transient or permanent reduction in CBF induced by an embolus or thrombosis blocking regional arteries (except in cases of lacunar infarction) (Dirnagl et al., 1999); it induces a series of pathological events, including BBB disruption, vasogenic edema, hemorrhagic transformation (HT), and neuronal injury (Sifat et al., 2017). Intravenous tissue-type plasminogen activator (tPA) is an effective treatment for AIS if administered within a specific therapeutic time window (Powers et al., 2015). However, BBB impairment restricts the application of this thrombolytic treatment; tPA passes through the impaired BBB and activates matrix metalloproteinase-9 (MMP-9) within the parenchyma, inducing ECM degradation and further altering BBB permeability. Consequently, BBB disruption aggravates cerebral edema, elevates risk of fatal HT, and intensifies the neuroinflammatory reaction after AIS (Khatri et al., 2012; Cheng et al., 2014). Therefore, BBB disruption is critical in AIS and contributes to subsequent brain damage.

Stroke is composed of two successive phases, ischemia and reperfusion, based on the paracellular penetrative state (Sandoval and Witt, 2008). In the ischemic phase, CBF is reduced, resulting in a deficient supply of glucose and oxygen (Sandoval and Witt, 2008). Glucose and oxygen are essential to maintain an adequate supply of adenosine triphosphate (ATP) to guarantee physiological cellular function and maintain normal ion gradients (Abdullahi et al., 2018). Following the onset of ischemia, oxidative phosphorylation is discontinued, followed by an ATP shortage (Hata et al., 2000). EC ion transporters are unable to function with inactivation of Na^+^-K^+^-ATPases and Ca^2+^-ATPases due to lack of energy. Increased activity of other ion transporters, such as Na^+^-H^+^ exchangers, NKCC1, and the calcium-activated potassium channel, KCa3.1, elevates intracellular Na^+^ levels and causes cytotoxic edema (Khatri et al., 2012; Xiaoyan et al., 2018). Na^+^ accumulation also leads to membrane depolarization and Na^+^-Ca^2+^ exchanger dysfunction, resulting in Ca^2+^ efflux and calcium overload (Abdullahi et al., 2018). This promotes the release of excitatory neurotransmitters and reactive oxygen species (ROS), which induce inflammatory cascades and destroy the BBB (Janardhan and Qureshi, 2004; Adibhatla and Hatcher, 2008). KCa3.1 blockade is capable of reversing cerebral edema within 3 h of AIS (Chen et al., 2015). Similarly, inhibition of NKCC1 can alleviate brain edema and neurological impairment (O’Donnell et al., 2004). Sodium-dependent glucose transporters (SGLTs) and GLUT1 both play a role in glucose transportation under pathological and physiological conditions, respectively. The function of SGLTs are dependent on the NKCC1-mediated sodium gradient, which contributes to sodium accumulation in ECs during stroke. Inhibition of SGLTs has been shown to reduce edema and improve poststroke outcome (Vemula et al., 2009). Interestingly, another study has shown that SGLTs are also present in neurons but not BBB ECs under physiological conditions (Yu et al., 2010; Patching, 2017). In any event, BBB ion transporters provide a hopeful therapeutic target.

**FIGURE 2:**
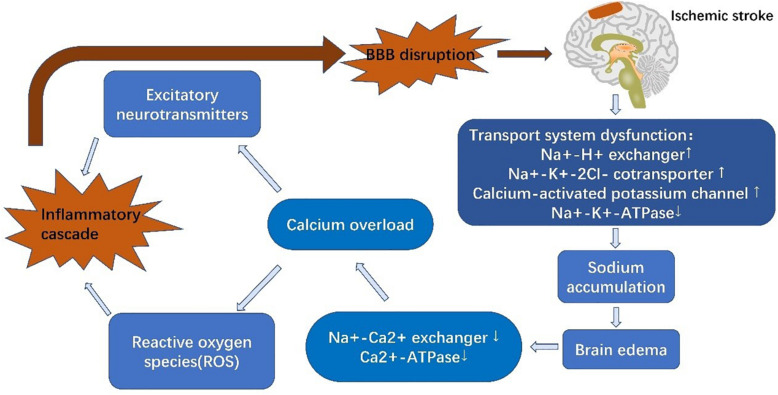
Schematic diagram of blood-brain barrier alterations in acute ischemic stroke. Reduced levels of glucose and oxygen result in dysfunction of EC ion transporters, inducing calcium overload and triggering inflammatory cascades. These inflammatory cascades further degrade tight junctions, thus aggravating brain edema and increasing infarct size.

In the reperfusion phase, CBF starts to recover, which is beneficial for neuronal survival but increases the risk of HT. BBB injury is a core pathophysiological mechanism in reperfusion damage (Vemula et al., 2009), and increased BBB permeability further increases HT risk (Sandoval and Witt, 2008). BBB dysfunction can be characterized into three stages: stage 1 includes the effects of oxidative stress on the BBB and associated ECM degeneration, whereas stages 2 and 3 are associated with vasogenic edema and angiogenesis. Alterations in BBB TJs during phase 1 induce the changes seen in stages 2 and 3 (Heo et al., 2005; Khatri et al., 2012). Activation of proteinases, such as MMPs, and tPA, are the most important contributors to BBB breakdown and are stimulated by either hypoxia-inducible factor-1α (HIF-1α)-dependent mechanisms or cytokines, such as tumor necrosis factor-α (TNF-α) and interleukin 1β (IL-1β) (Yang and Rosenberg, 2011). MMPs, particularly MMP-2 and MMP-9 have been shown to degrade TJ proteins (Yang and Rosenberg, 2011; Liu et al., 2012). MMP-9, derived from neutrophils, degrades claudin-5 and the BM following ischemia, resulting in brain edema, neurological deficits and increased HT risk (McColl et al., 2008). In experimental stroke mice, elevated levels of α2-antiplasmin enhanced the expression of MMP-9, exacerbating BBB breakdown and ischemic injury (Singh et al., 2018). In addition to MMPs, caveolin-1 mediates the translocation and downregulation of claudin-5, exacerbating BBB leakage (Liang et al., 2015). Furthermore, exudation of inflammatory cells also aggravates BBB breakdown (Sifat et al., 2017), accompanied by reduced expression of occluding (Liang et al., 2015), ZO-1 (Jiao et al., 2011), and VE-cadherin (Wacker et al., 2012; Weinl et al., 2015). For example, microglia secrete cytokines to stimulate upregulation of intercellular adhesion molecule-1 (ICAM-1), *P*-selectin and *E*-selectin on the surface of ECs, mediating the infiltration of neutrophils (Wang and Doerschuk, 2002). Rho kinase (RhoK) mediates the phosphorylation of occludin and claudin-5, increasing BBB permeability (Yamamoto et al., 2008). C-C motif ligand 2 (CCL2), also known as monocyte chemoattractant protein-1 (MCP-1) elevates expression of caveolin-1, resulting in phosphorylation of occludin and ZO-1, and thus, exacerbating BBB leakage. In summary, ischemia and hypoxia induce BBB dysfunction, which then facilitates inflammatory cascades that further induce decompose BBB dysfunction. Thus, the BBB is not only influenced by AIS but also contributes to AIS pathology (Figure 2).

In recent years, research has focused on exploring advanced therapies to alleviate BBB damage. Chemokine receptor type 5 (CCR5) stimulates regulatory T cells (Tregs) strengthens BBB immunoregulatory function and ameliorates inflammatory cascades, thus representing a potential treatment target (Li et al., 2017). Circular RNA DLGAP4 (circDLGAP4) can bind to miR-143, maintaining BBB integrity and diminishing infarct volume in AIS patients (Bai et al., 2018). miR-98 protects EC stability and strengthens BBB integrity through modifying activation of small Rho GTPases, rearranging the actin cytoskeleton, and redistributing TJ proteins. It also limits the recruitment of inflammatory cells by reducing expression of the proinflammatory cytokines, CCL2 and CCL5 (Bernstein et al., 2019). Transplantation of mesenchymal stem cells (MSCs) is a prominent research focus as they can promote neurogenesis and angiogenesis in the BBB (Dabrowska et al., 2019). Integrin α5β1 mediates leukocyte infiltration and subsequent BBB dysfunction; the integrin α5β1 inhibitor, ATN-161, can preserve claudin-5 and collagen-IV expression and reduce MMP-9 transcription, thus protecting BBB integrity (Edwards et al., 2019). Perlecan, a major heparan sulfate proteoglycan component of the BM, can maintain and repair the injured BBB by reinforcing the BM (Nakamura et al., 2019). Furthermore, release of microvesicles by ECs can upregulate ZO-1 and claudin-5 expression, thereby ameliorating BBB disruption and reducing infarct volume (Pan et al., 2016). Cystatin C is also capable of reducing the expression of caveolin-1, restricting the activity of MMP-9 and elevating occludin expression in mice that have undergone middle cerebral artery occlusion (MCAO) (Yang et al., 2019). It has been shown that deficiency of vitamin D hormone (VDH) reduces occludin and claudin-5 expression and correlates with poor prognosis in stroke (Balden et al., 2012; Stessman and Peeples, 2018). Therefore, sufficient supplementation of VDH appears to be advantageous to maintaining BBB integrity (Sayeed et al., 2019). Nanoparticles are an efficient method for transporting pharmacological compounds across the BBB. For instance, Bao et al. (2018) designed edaravone-loaded ceria nanoparticles that cleared ROS produced by AIS and effectively protected the BBB with minimal side-effects. Combination treatment with recombinant tissue plasminogen activator (rt-PA) and minocycline or rt-PA and cilostazol has been shown to protect TJ proteins from secondary degradation following thrombolysis (Ishiguro et al., 2010; Fan et al., 2013). Moreover, type 5 NADPH oxidase (NOX5) induces ROS; thus, applying a NOX5 inhibitor together with reperfusion treatment (thrombolysis or thrombectomy) has been shown to reduce ROS generation and ameliorate BBB injury (Casas et al., 2019). CO-releasing molecule (CORM)-3 decreases the level of TNF-α, and IL-1β, upregulates expression of PDGFR-β and ZO-1 and inhibits activity of MMP-9, thus alleviating BBB disruption in transient-MCAO mice (Wang et al., 2018).

### Intracerebral Hemorrhage

Intracerebral hemorrhage (ICH) is a leakage of blood components from damaged vessels and into the brain parenchyma (Qureshi et al., 2009). The pathophysiology of ICH includes two phases; hematomas induce the initial mechanical compression damage, which leads to mitochondrial dysfunction and membrane depolarization. Cells affected by this damage induce secondary injury, which is characterized by excitotoxicity and oxidative stress caused by cellular dysfunction and the presence of blood components (Qureshi et al., 2009; Shi et al., 2019). Perihematomal edema (PHE) is evident in both stages although the relevant pathogenesis is different.

PHE develops immediately after ICH onset (Venkatasubramanian et al., 2011), which leads to rapid deterioration in the patient and subsequent poor prognosis (Mayer et al., 1994). It can be divided into three phases: phase 1 includes osmotic edema caused by the initial damage, and phases 2 and 3 are characterized by vasogenic edema resulting from secondary damage (Urday et al., 2015).

**FIGURE 3:**
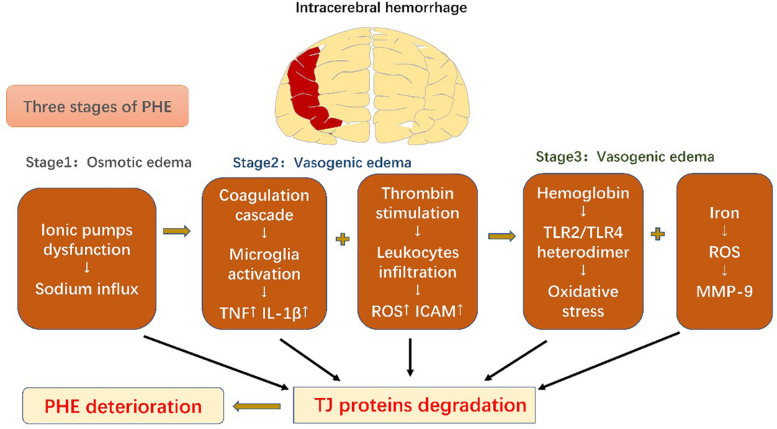
Schematic diagram of blood-brain barrier changes in intracerebral hemorrhage. Perihematomal edema secondary to intracerebral hemorrhage can be divided into three phases. All the pathophysiological events result in degradation of tight-junction proteins, which exacerbate perihematomal edema and result in poor prognosis.

The osmotic edema phase, phase 1, occurs within the first hours of ICH onset and is composed of clot retraction and cytotoxic edema (Xi et al., 2002). Clot retraction occurs due to a secondary coagulation cascade, which extrudes serum proteins and prompts interstitial accumulation of ions and water. The cytotoxic edema can mainly be ascribed to dysfunction of cellular ionic pumps (such as Na^+^-K^+^-ATPase and NKCC1), resulting in sodium influx and edema formation (Urday et al., 2015). Phase 2, including the first 2 days in ICH, encompasses a further coagulation cascade and stimulation of thrombin. Thrombin combines with protease-activated receptor-4 (PAR-4) and stimulates microglia (Bodmer et al., 2012), which then release cytokines including TNF and IL-1β; TNF has been shown to be a pivotal factor in downregulation of the TJ proteins (Xi et al., 2002; Aslam et al., 2012). However, thrombin also induces PAR-1 and phosphorylates myosin light chain (MLC) in ECs, strengthening their contractility and increasing intercellular gaps (Simard et al., 2010; Urday et al., 2015). Moreover, thrombin facilitates leukocyte infiltration, followed by ROS production and increased ICAM expression in ECs, which further enhances leukocyte exudation (Bijli et al., 2007). The complement cascade also contributes to PHE development. Recruitment of chemokines results in generation of membrane attack complexes (MACs) that promote dissolution of red blood cells (RBCs) and edema induced by blood components (Bodmer et al., 2012; Urday et al., 2015). Phase 3, following from phase 2, also contributes to BBB disruption. RBCs are degraded into hemoglobin (Hb) and iron by MACs. Hb activates the Toll-like receptor-2/4 (TLR2/TLR4) heterodimer, which promotes oxidative stress and mediates further BBB disruption (Urday et al., 2015). MMP-9, as a downstream factor activated by ROS, is key to iron-mediated edema. As previously discussed, it degrades TJ proteins and the basal lamina to aggravate BBB leakage (Florczak-Rzepka et al., 2012). Conventional treatment for ICH includes respiratory support, management of both blood and intracranial pressures, anti-coagulation therapy, and surgical evacuation (Qureshi et al., 2009). The BBB serves as a trigger point for inflammatory cascades and, thus, participates in the whole ICH disease course. Considering this central role, advanced treatments have recently been developed that aim to modulate this immunoreaction to reduce secondary BBB injury of BBB (Figure 3).

Statins can alleviate PHE in ICH through anti-inflammatory mechanisms. Siddiqui et al. (2017) analyzed the use of statins in ICH patients and found that patients taking statins had better clinical outcomes. Likewise, celecoxib can inhibit inflammation and reduce brain edema through lowering prostaglandin E2 expression (Chu et al., 2004; Lee et al., 2013). The colony-stimulating factor 1 receptor inhibitor, PLX3397 depletes microglia, inhibits leukocyte infiltration and decreases the level of pro-inflammatory mediators, thereby eliminating BBB breakdown and brain edema (Li et al., 2016). Transforming growth factor-β1 (TGF-β1) has also been shown to improve ICH prognosis through modulation of microglial phenotype and inhibition microglial-derived neuroinflammation (Taylor et al., 2017). LRP-1 is able to obliterate hemoglobin, blocking subsequent damage and, thus, stabilizing the BBB in mice (Wang et al., 2016). Fingolimod, an immunomodulatory compound, has also been shown to protect the BBB and improve clinical sequelae through reducing MMP-9 expression, inhibiting infiltration of immune cells and secretion of inflammatory mediators (Rolland et al., 2013; Li et al., 2015). The peroxisome proliferator-activated receptor γ (PPAR-γ) agonist, pioglitazone, is in phase II clinical trials to evaluate the safety of its application to promoting erythrocyte phagocytosis by microglia, which alleviates inflammation and protects the BBB *in vivo* (NCT00827892) (Fu et al., 2015). In addition, a randomized pilot phase II clinical trial of minocycline is also (NCT01805895) (Fu et al., 2015) assessing its BBB protection ability via reducing microglial infiltration and downregulating pro-inflammatory cytokine expression (Wu et al., 2010).

## Neurodegenerative Disease

### Alzheimer’s Disease

Alzheimer’s disease is characterized by deposition of misfolded Aβ and tau proteins in the CNS parenchyma (Hardy and Selkoe, 2002), leading to BBB breakdown and cognitive impairment (Montagne et al., 2015; Jevtic et al., 2017). Autopsy studies on patients with confirmed AD showed that more than half of these patients had evident vascular alterations (Sweeney et al., 2019a). Hereditary susceptibility combined with environmental factors, such as pollutants and diet, evoke a series of vascular changes. In fact, BBB disruption, as a contributor to the pathophysiological process, exists before the formation of typical vascular pathology. There is two-hit vascular hypothesis (Nelson et al., 2016), in which vascular dysfunction plays a leading role in the initial stages, resulting in ischemia-hypoxia and EC injury, and then facilitates deposition of neurotoxic substances in the CNS during the second stage.

APOE genetic variants, among the greatest genetic risk factors for sporadic AD, have been shown to cause BBB disruption and degeneration of PCs (Verghese et al., 2011; Halliday et al., 2016). In APOE4 carriers with normal cognition or mild cognitive impairment (MCI), a prelude to AD, dynamic contrast-enhanced magnetic resonance imaging evidenced BBB leakage prior to tissue loss, indicating that BBB disruption is independent of Aβ and tau deposition (Ishii and Iadecola, 2020; Montagne et al., 2020). In addition, secreted PDGFRβ (sPDGFRβ), a biomarker of PC injury, is elevated in the cerebrospinal fluid (CSF) of APOE4 carriers (Ishii and Iadecola, 2020; Montagne et al., 2020) and has been shown to be independent of pathological changes in AD (Montagne et al., 2020). Of all APOE isoforms, APOE4 increases AD susceptibility dramatically compared with APOE3, and APOE2 reduces AD risk (Verghese et al., 2011). APOE4 activates cytokine cyclophilin A (CypA), nuclear factor-κB (NF-κB) and MMP-9 in both PCs and ECs, degrading TJ and BM proteins, downregulating GLUT1 expression and upregulating expression of RAGE (Montagne et al., 2017). RAGE binds peptides and mediates influx of Aβ (Candela et al., 2010), thus damaging PCs and the BBB (Bell et al., 2012; Halliday et al., 2016). The impact of APOE4 on ECs and PCs is independent of Aβ or tau elevation (Ishii and Iadecola, 2020). Furthermore, increased APOE burden reduces Aβ transport, resulting in its accumulation in the brain and accelerating the course of the disease (Gosselet, 2012). Therefore, BBB dysfunction can independently trigger subsequent disorders and exacerbate cognitive impairment.

Moreover, evidence indicates that VSMCs and ECs can produce Aβ peptides in the adult brain (Kitazume et al., 2010; Schweinzer et al., 2011). The BBB is also an underlying source of Aβ peptides, which downregulate ZO-1, occludin, and claudin-5 and increase the permeability of ECs through interaction with RAGE, activation of MMPs and stimulation of oxidative stress pathways (Carrano et al., 2011; Kook et al., 2012). In addition, it has been shown that Aβ peptides promote angiogenesis and redistribution of TJs, impairing BBB integrity and allowing Aβ leakage (Biron et al., 2011).

The ABC superfamily is important for lipoprotein metabolism and Aβ efflux (Kim et al., 2008; Miller, 2015). *P*-gp and ABCG2 not only facilitate transport of Aβ across the BBB via LRP1 binding (Cirrito et al., 2005; Storck et al., 2018), but also restrict Aβ influx (Candela et al., 2010). ABCA1 mediates the transfer of cholesterol to APOE, high-density lipoprotein (HDL), and APOA-I and modifies the cleavage of amyloid precursor protein (APP) as well as clearance of Aβ across the BBB (Saint-Pol et al., 2013; Kuntz et al., 2015; Dal Magro et al., 2019). ABCA7 is the only ABC transporter implicated by AD Genome Wide Association Studies (GWAS) (Hollingworth et al., 2011). Absence of ABCA7 expression reduces the level of ABCA1 and APOE secretion with a concurrent reduction in cholesterol exchange (particularly that of HDL and ApoA-I) and Aβ efflux (Lamartiniere et al., 2018).

**FIGURE 4:**
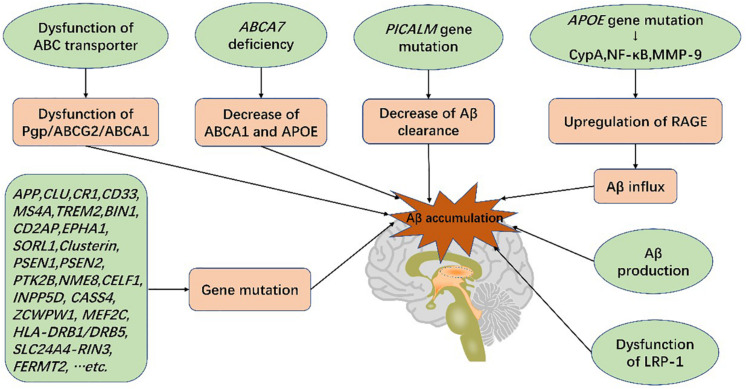
Genetic mutations and Aβ production in the blood-brain barrier increase the risk of AD. Genetic mutations, such as ABCA7, PICALM, and APOE, lead to dysfunction of BBB transport systems, resulting in Aβ deposition in the brain. Aβ accumulation gives rise to blood-brain barrier breakdown and cognitive impairment, characteristic of Alzheimer’s disease.

LRP-1 is the main transporter of Aβ with dysfunction increasing Aβ levels and promoting AD (Jaeger et al., 2009). *P*-gp, located on the luminal side of ECs, limits the transport of multiple products (Schinkel et al., 1994), including Aβ. *P*-gp deficiency contributes to LRP1 dysfunction and increases Aβ levels (Cirrito et al., 2005). A recent experiment involved in the BBB model *in vitro* indicated that a ketogenic diet could enhance expressions of LRP1, *P*-gp, and PICALM and, thus, promote Aβ efflux across the BBB (Versele et al., 2020). PICALM has been shown to promote binding of LRP1 to Aβ as well. It promotes Aβ endocytosis and Aβ-LRP1 transcytosis by transferring the protein complex to Rab5 and Rab11 (Zhao et al., 2015b). Furthermore, PICALM gene mutations have also been implicated in AD (Tian et al., 2013; Zhao et al., 2015a) (Figure 4).

Therapeutic BBB modulation has shown promising results in AD treatment. FPS-ZM1, an innoxious RAGE suppressant, inhibits RAGE activation and reduces entry of Aβ within the brain, thereby suppressing neuroinflammation (Deane et al., 2012). Van Skike et al. (2018) found that inhibiting the mammalian/mechanistic target of the rapamycin (mTOR) pathway can maintain BBB integrity and restore cognitive function. Bana et al. (2014) also succeeded in constructing a nanodevice to alter Aβ morphology and treat AD, which needs further clinical trials.

### Parkinson’s Disease

Parkinson’s disease (PD) is a neurodegenerative disease characterized by tremors, limb rigidity, and hypokinesis (Calne and Sandler, 1970). The main pathological hallmark of PD is the progressive death of dopaminergic neurons in the substantia nigra (SN) and deposition of Lewy bodies (LBs), fibrillar aggregates composed of α-synuclein (α-syn), leucine-rich repeat kinase 2 (LRRK2), and other proteins (Lang and Lozano, 1998; Wakabayashi et al., 2013). PD occurrence is influenced by hereditary susceptibility, environmental toxins (such as rotenone and pesticides), age-related neurodegeneration, and interactions between these factors.

BBB lesions, including a thickened BM and degraded PCs, have been observed in PD (Farkas et al., 2000). Additionally, prominent BBB leakage has been shown in the striatum of PD patients with deposition of fibrin and hemosiderin forecasting its vulnerability (Gray and Woulfe, 2015). Positive correlations have also been found between parenchymal iron levels, resulting from BBB leakage, and brain pathology in PD patients (Götz et al., 2004). In addition to iron, extravasation of red blood cells that act as a source of α-syn might also contribute to PD pathology (Sweeney et al., 2018). Degradation of TJ proteins cause BBB injury and enhanced transcytosis. A recent study showed that misfolded α-syn could downregulate ZO-1 and occludin expression, indicating that α-syn is neurotoxic to the BBB (Kuan et al., 2016). Degradation of ECs also generates BBB breakdown and initiates neurodegenerative pathology. In addition, decreased expression of GLUT-1 and ABCG2 were evident in PD mouse models, and P-gp expression was increased (Vautier et al., 2009a; Sarkar et al., 2014). However, another study has found a reduction in *P*-gp expression although this could be attributed to the methods of establishing the models (Huang et al., 2016; Pan and Nicolazzo, 2018). *P*-gp dysfunction also plays an important role in PD pathogenesis due to decreased clearance of neurotoxic substances (Kortekaas et al., 2005), leading to their accumulation in the BBB, similarly to its role in AD pathogenesis. Thus, P-gp could be a promising therapeutic target in PD as well. Despite clearly thickened BM and decreased *P*-gp function in PD (Pan and Nicolazzo, 2018), the specific pathological mechanisms remain unclear. Neuroinflammation likely also plays a crucial role in this process with α-syn transport or iron deposition initiating the inflammatory reaction. It has been shown that metabolic iron disorders induce oxidative stress and free radical production, resulting in the induction of neurotoxic cascades (Crichton et al., 2011; Weinreb et al., 2013). Activated microglia secrete inflammatory factors, upregulate EC expression of adhesion molecules and recruit leukocytes, and amplify inflammatory cascades, ultimately resulting in neuronal (Reale et al., 2009). Inflammatory cytokines also downregulate ZO-1 and occludin, thus increasing BBB permeability (Wong et al., 2004). The inflammatory mediators MMP-3 and nuclear factor erythroid 2–related factor 2 (NRF2) have been show to stimulate α-Syn production, induce aggregation of amyloidogenic compounds and induce the release of inflammatory cytokines, mediating death of dopaminergic neurons (Rivera et al., 2019; Sivandzade et al., 2019). Interestingly, knockout of TNF-α expression in PD model mice attenuated BBB leakage (Zhao et al., 2007).

Furthermore, CSF from PD patients contains elevated levels of angiogenesis factors (Janelidze et al., 2015). Aberrant angiogenesis results in generation of immature vasculature and loose intercellular junctions, which has been connected with dopaminergic neuronal loss through leakage of neurotoxic substances (Desai et al., 2007). To summarize, BBB injury leads to substantial leakage of peripheral substances, thus expediting development of neurodegenerative disorders.

There are now multiple advanced treatments and newfound targets for PD. Milk fat globule-epidermal growth factor-VIII (MFG-E8) can promote neurogenesis and restore impaired neurocytes in PD (Cheyuo et al., 2019). Statins exert neuroprotective effects in PD models through anti-inflammatory and anti-oxidation mechanisms, providing a promising treatment option (Roy and Pahan, 2011; Wood et al., 2014). Glutathione is an antioxidative agent and demonstrates beneficial effects in PD by protecting dopaminergic neurons (Smeyne and Smeyne, 2013). Anti-complement agents that regulate immunoreactivity through inhibition of complement pathways have been effective in PD models but require further verification in clinical trials (Carpanini et al., 2019). Increased expression of hepcidin accelerates iron transport and metabolism, thus providing an additional potential therapy (Qian and Ke, 2019). Iron-chelation therapy is beneficial for adjusting iron homeostasis (Weinreb et al., 2013). Glial cell-derived neurotrophic factor (GDNF), nerve growth factor (NGF), and brain-derived neurotrophic factor (BDNF) are all beneficial for neural revival and regeneration, thus transporting these factors across the BBB could be an option for PD treatment (Allen et al., 2013). Several options are available for improved delivery of therapeutics across the BBB, including using micro- or nanotechnology to bind therapeutic agents or delivery of small hydrophobic agents with liposomal targeting (Torres-Ortega et al., 2019; Wang et al., 2019). Focused ultrasound (FUS) to open specific BBB regions and facilitate entry of therapeutic compounds is currently in phase 1 trials for PD treatment (NCT03608553) (Zlokovic, 2008).

### Multiple Sclerosis

Multiple sclerosis (MS) is characterized by remitting-relapsing demyelination and axon loss. It is a chronic and progressive CNS inflammatory disorder, which ultimately evolves over time into neurodegeneration (Hemmer et al., 2002). The use of gadolinium enhancement in MRI has allowed measurement of BBB breakdown in MS (Miller et al., 1998), which is deemed as a trigger point for disease onset; the presence of immunocytes in the immune-privileged brain parenchyma via BBB breakdown induces the autoimmune disease in a hereditarily susceptible population (Daneman, 2012).

Energy metabolism dysfunction and endocrine disorders have been shown to initiate MS through crosstalk with immune cells (Procaccini et al., 2014; De Rosa et al., 2015). In addition, viral infection and environmental toxins in hereditarily susceptible individuals have also been shown to reduce immune tolerance and stimulate release of proinflammatory factors, such as IL-6 and NF-κB. As previously mentioned, these proinflammatory factors promote alternation of TJs and damage BBB integrity (Weksler et al., 2005) as well as facilitate leukocyte transmigration (Sheikh et al., 2020). *P*-gp expression is also increased and enhances migration of CD4^+^ and CD8^+^ T cells to further amplify neuroinflammation (Kooij et al., 2014). Silencing of P-gp expression significantly reduces CD8^+^ T cell trafficking into the CNS (Kooij et al., 2014). Under inflammatory conditions, EC expression of selectins and their corresponding ligands are also elevated, promoting leukocyte binding; this mediates further interaction between integrins and their ligands, thus strengthening the adherence of leukocytes (Wilson et al., 2010). Further to this, cytokines and chemokines upregulate endothelial adhesion receptors, including PECAM1, *E*-selectin, VCAM, and ICAM-1 and augment subsequent leukocyte infiltration (Ricci et al., 2009). PECAM-1 facilitates leukocyte adherence and directs para-endothelial infiltration, thereby contributing to increased inflammation (Ricci et al., 2009; Losy, 2013). VCAM upregulates the expression of integrin α-4 in ECs, reducing the level of TJ proteins (Haarmann et al., 2015). Integrin α-4 is also indispensable for CD8 + T lymphocyte recruitment (Ifergan et al., 2011). ICAM-1 plays a crucial role in inflammation through phosphorylation of TJ proteins and restructuring of the cytoskeleton, thus promoting increased BBB permeability (Huber et al., 2001). Both VCAM and ICAM mediate trans-endothelial infiltration of leukocytes (Hernández-Pedro et al., 2013). CD4^+^ T cells identify myelin sheath proteins and macrophages, and upregulate the release of pro-inflammatory factors (including IFN-γ, TNF-α, nitric oxide and free radicals), thus leading to demyelination (Hernández-Pedro et al., 2013). Neutrophils, monocytes, and microglia infiltrate the brain parenchyma and release extracellular glutamate, generating excitotoxity and accelerating BBB dysfunction (Macrez et al., 2016). Furthermore, fibrinogen leakage from the BBB prompts release of ROS by microglia (Davalos et al., 2012). Accumulation of leukocytes in the parenchyma induces further release of ROS, resulting in demyelination and breakdown of TJ proteins (Van der Goes et al., 2001; Hendriks et al., 2005). Demyelinated lesions further induce secretion of free radicals, magnifying the inflammatory reaction (Bagasra et al., 1995). As a result, the excessive oxidative stress downregulates occludin and ZO-1, thus further promoting TJ disruption (Ortiz et al., 2014).

Other members of the NVU also disrupt BBB integrity. In pathological circumstances, the AQP4-mediated polarization of ACs becomes misregulated, resulting in retraction of end-feet from the glia limitans (Wolburg-Buchholz et al., 2009; Spencer et al., 2018). Moreover, AC-expressed VEGF-A activates the downstream factor, *e*-nitrogen oxide (eNOS), which disrupts EC expression of claudin-5 and occludin and disrupts the BBB (Argaw et al., 2012). Oligodendrocyte precursor cells (OPCs) also accumulate in MS lesions, interrupting AC end-feet contacts and TJ integrity as well as impairing lesion repair and altering BBB permeability (Niu et al., 2019). To summarize, immune system abnormalities induce alterations in BBB TJs, which facilitates trans-endothelial migration of leukocytes and ultimately leads to neurodegeneration (Sheikh et al., 2020).

Disease-modifying therapies (DMTs) are a basic therapy for MS with multiple advanced drugs developed and clinically tested. Ozanimod, a sphingosine 1-phosphate (S1P) receptor modulator, was approved for MS treatment in March 2020 (Lamb, 2020). Siponimod is undergoing a phase-3 trial (NCT01185821) due to its beneficial effects on relapsing-remitting MS (Kappos et al., 2016). Ibudilast is currently in phase-2 trials due to its capability of crossing the BBB and restricting the shift in macrophage phenotype, thus benefiting progressive MS (NCT01982942) (Fox et al., 2018). Immunoablation and autologous hemopoietic stem-cell transplantation (aHSCT) has passed a phase 2 single-arm trial, which aims to provide long-term control of aggressive MS (NCT01099930) (Atkins et al., 2016). Minocycline has also been verified in clinical trials to lower the risk of clinically isolated syndromes progressing into MS (NCT00666887) (Metz et al., 2017). As previously stated, application of nanomaterials is an efficient way to deliver agents across the BBB (Furtado et al., 2018). Fingolimod reduces monocyte migration and reverses BBB dysfunction through targeting ACs (van Doorn et al., 2012). However, a recent large comparative study found that patients treated with fingolimod have a higher risk of cancer due to increased tumorigenicity from immunosuppression; use of rituximab and natalizumab in MS do not elevate invasive cancer risk (Alping et al., 2020). Natalizumab, a monoclonal antibody (McAb) mainly used in treatment of relapsing-remitting MS, can prevent leukocyte adherence and blocks integrin α-4 on ECs, thereby protecting the BBB (Haarmann et al., 2015). However, as an efficient inflammatory suppressant, it also inhibits immune surveillance and leads to progressive multifocal leukoencephalopathy (PML), an infectious disease caused by John Cunningham virus (Prezioso et al., 2020), which unfortunately restricts its clinical application.

## Conclusion

In summary, BBB disruption plays a central role in the pathophysiology of cerebrovascular and neurodegenerative disease. Dysfunction in energy metabolism and leakage of blood components catalyze CNS inflammation in cerebrovascular disease. Conversely, in neurodegenerative disease, endogenous inflammation is stimulated when genetically susceptible individuals develop endocrine disorders or are exposed to environmental factors, such as pollutants and exogenous infection. Despite diverging induction mechanisms, inflammation is a common characteristic of these CNS diseases. In general, disruption of the homeostatic CNS microenvironment triggers BBB reconstruction, resulting in leukocyte infiltration and leakage of neurotoxic substances that augment further neuroinflammation, which, in turn, exacerbates BBB permeability. Therefore, preservation of the BBB is a crucial therapeutic target. Recent investments mainly focus on the following two aspects: modulation of the inflammatory cascade modulation (including NOS, ROS, cytokines and chemokines) and BBB reinforcement. In this review, we have first correlated pro-inflammatory mediators with changes in the corresponding BBB-expressed molecules and listed several recent advanced treatments targeting the BBB and the inflammatory response. Existing therapies are also being applied to limit severe BBB dysfunction and the inflammation cascade. Further studies should concentrate on limiting the initial inflammation prior to BBB remodeling and on consolidating BBB integrity by directly promoting the synthesis of relevant TJ proteins; this may significantly improve treatment efficacy. In a word, BBB dysfunction is more of a contributor to CNS pathology than a victim in neurological disorders, and so more attention should be paid to its significant function in maintaining CNS homeostasis.

## Author Contributions

MX: study concept, study design, manuscript preparation, and editing. ZX: literature research and manuscript revision. BY: manuscript revision. ZL: literature research and management. FF: figure design. All authors contributed to the article and approved the submitted version.

## Conflict of Interest

The authors declare that the research was conducted in the absence of any commercial or financial relationships that could be construed as a potential conflict of interest.

## Abbreviations

A β: amyloid β-peptide
ABC: active efflux-ATP-binding cassette
AD: Alzheimer’s disease
aHSCT: autologous hemopoietic stem-cell transplantation
AIS: acute ischemic stroke
AJs: adherence junctions
Ang-1: angiopoietin-1
APOE: apolipoprotein E
APP: β-amyloid precursor protein
AQP-4: aquaporin-4
ATP: adenosine triphosphate
BBB: blood-brain barrier
BDNF: brain-derived neurotrophic factor
BM: basement membrane
BMPs: bone morphogenetic proteins
CBF: cerebral blood flow
CCL2: (C-C motif) ligand 2
CCR5: chemokine receptor type 5
CMT: solute carrier-mediated transport
CNS: central nervous system
CSF: cerebrospinal fluid
CSF: cerebrospinal fluid
CypA: cytokine cyclophilin A
DMTs: disease-modifying treatments
ECM: extracellular matrix
ECs: endothelial cells
ESAM: endothelial cell adhesion molecule
FUS: focused ultrasound
GDNF: glial cell-derived neurotrophic factor
GLUT1: glucose transporter-1
Glut1-DS: glucose transporter-1 deficiency syndrome
GWAS: Genome Wide Association Study
HDL: high-density lipoprotein
Hh: Hedgehog
HT: hemorrhagic transformation
ICAM-1: adhesion molecule-1
ICAM-1: intercellular adhesion molecule-1
ICH: intracerebral hemorrhage
IL-1 β: interleukin 1 β
JAMs: junctional adhesion molecules
LBs: Lewy bodies
LRP: lipoprotein receptor–related protein
LRRK2: leucine-rich repeat kinase 2
MAGUK: membrane-associated guanylate kinases
McAb: monoclonal antibody
MCI: mild cognitive impairment
MLC: myosin light chain
MMP-9: matrix metalloproteinase-9
MS: multiple sclerosis
NF- κ B: nuclear factor- κ B
NGF: neurotrophin nerve growth factor
NOS: nitric oxide synthase
NVU: neurovascular unit
OGD: oxygen-glucose deprivation
OPC: oligodendrocyte precursor cells
PAR-4: protease-activated receptor-4
PCs: pericytes
PD: Parkinson’s disease
PDGF-BB: platelet-derived growth factor BB
*P*-gp: *P*-glycoprotein
PDGFR β: platelet-derived growth factor-receptor- β
PECAM-1: platelet endothelial cell adhesion molecule-1
PICALM: phosphatidylinositol-binding clathrin assembly
PML: progressive multifocal leukoencephalopathy
PPAR- γ: Peroxisome proliferator-activated receptor γ
RA: retinoic acid
RAGE: receptor for advanced glycation end products
RhoK: Rho kinase
RMT: receptor-mediated transport
ROS: reactive oxygen species
rt-PA: recombinant tissue plasminogen activator
S1P: sphingosine 1-phosphate
SGLT: sodium-dependent glucose transporters
SHH: Sonic hedgehog
Smad: Sma- and Mad-related protein
SN: substantia nigra
TGF- β 1: Transforming growth factor- β 1
TJ: tight junction
TNF- α: tumor necrosis factor α
tPA: tissue-type plasminogen activator
Tregs: regulatory T cells
VCAM: vascular cell adhesion molecules
VEGF: vascular endothelial growth factor
VSMC: vascular smooth muscle cell
ZO: zonula occludens.
